# Insight into the Role of Dental Pulp Stem Cells in Regenerative Therapy

**DOI:** 10.3390/biology9070160

**Published:** 2020-07-09

**Authors:** Shinichiro Yoshida, Atsushi Tomokiyo, Daigaku Hasegawa, Sayuri Hamano, Hideki Sugii, Hidefumi Maeda

**Affiliations:** 1 Department of Endodontology, Kyushu University Hospital, 3-1-1 Maidashi, Higashi-ku, Fukuoka 812-8582, Japan; tomokiyo@dent.kyushu-u.ac.jp (A.T.); daigaku8@dent.kyushu-u.ac.jp (D.H.); sugii@dent.kyushu-u.ac.jp (H.S.); hide@dent.kyushu-u.ac.jp (H.M.); 2 OBT Research Center, Faculty of Dental Science, Kyushu University, 3-1-1 Maidashi, Higashi-ku, Fukuoka 812-8582, Japan; shamano@dent.kyushu-u.ac.jp; 3 Department of Endodontology and Operative Dentistry, Faculty of Dental Science, Kyushu University, 3-1-1 Maidashi, Higashi-ku, Fukuoka 812-8582, Japan

**Keywords:** dental pulp stem cells, stem cells from human exfoliated deciduous teeth, regenerative therapy, cell-based therapy, regenerative medicine

## Abstract

Mesenchymal stem cells (MSCs) have the capacity for self-renewal and multilineage differentiation potential, and are considered a promising cell population for cell-based therapy and tissue regeneration. MSCs are isolated from various organs including dental pulp, which originates from cranial neural crest-derived ectomesenchyme. Recently, dental pulp stem cells (DPSCs) and stem cells from human exfoliated deciduous teeth (SHEDs) have been isolated from dental pulp tissue of adult permanent teeth and deciduous teeth, respectively. Because of their MSC-like characteristics such as high growth capacity, multipotency, expression of MSC-related markers, and immunomodulatory effects, they are suggested to be an important cell source for tissue regeneration. Here, we review the features of these cells, their potential to regenerate damaged tissues, and the recently acquired understanding of their potential for clinical application in regenerative medicine.

## 1. Introduction

Mesenchymal stem cells (MSCs) have been reported to be isolated from various somatic tissues such as bone marrow [1], adipose tissue stromal vascular fraction [2], umbilical cord [3], and placenta [4]. They are noteworthy for their robust self-renewal capacity and multilineage differentiation potential including osteogenic, adipogenic, chondrogenic, myogenic, neurogenic, and tenogenic [5,6,7,8]. Thus, cell-based medicine using MSCs is expected to facilitate tissue regeneration in various somatic disorders. MSC-like populations have also been identified in dental tissues. Gronthos et al. initially identified dental stem cells from the human pulp tissue and termed dental pulp stem cells (DPSCs) [9]. Subsequently, dental MSC-like populations from other dental tissues were identified and characterized: Stem cells from human exfoliated deciduous teeth (SHEDs) [10], periodontal ligament stem cells (PDLSCs) [11], dental follicle precursor cells (DFPCs) [12], and stem cells from apical papilla (SCAPs) [13]. Among these stem cells, DPSCs and SHEDs are more attractive as cell sources for regenerative medicine than other MSC-like populations because of their easy accessibility.

**Figure 1:**
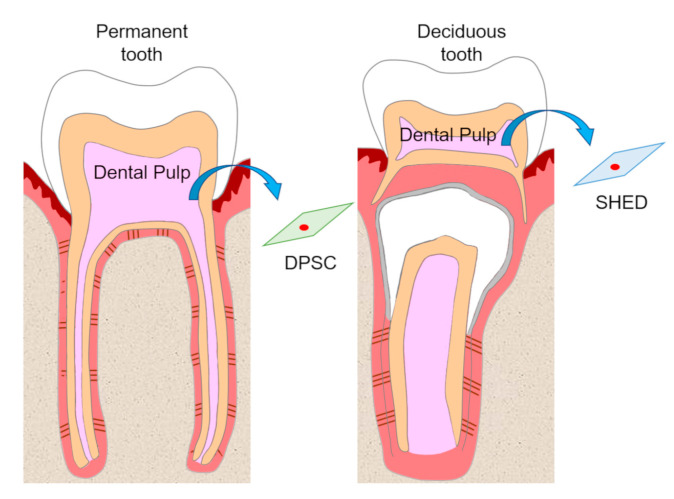
Isolation of dental pulp stem cells (DPSCs) and stem cells from human exfoliated deciduous teeth (SHEDs). DPSCs and SHEDs isolated from pulp tissue of permanent teeth and deciduous teeth, respectively, have the potential to be cell sources in regenerative medicine.

Dental pulp is an unmineralized connective tissue that is composed of stem cells as well as fibroblastic cells, capillary blood vessels, peripheral nerves, lymphatic elements, and extracellular matrices and odontoblasts on the periphery of pulp tissue. DPSCs are isolated from adult human pulp tissue of impacted third molars, orthodontic teeth, and supernumerary teeth, and have been characterized as cells with intense clonogenicity, proliferative activity, and the ability to form mineralized nodules [9] (Figure 1).

In addition, DPSCs have been clarified to possess multilineage differentiation potential and to express MSC-related markers [14,15,16,17]. Subsequently, the isolation of SHEDs was reported; these cells were characterized as having high proliferative activity and the ability to differentiate into osteoblasts, adipocytes, odontoblasts, and neural cells [10] (Figure 1). Like DPSCs, SHEDs are advantageous in terms of their ease of isolation in a minimally invasive manner compared with MSCs from other tissues. Specifically, DPSCs can easily be obtained from wisdom teeth and premolars from orthodontic treatment, while SHEDs can be isolated from primary teeth that are lost due to the eruption of permanent teeth. Given the expression of MSC-related markers, multipotency, origin, and therapeutic potential of DPSCs and SHEDs, they have attracted substantial attention in terms of their translation into clinical application. Because of their MSC-like characteristics, they are suggested to be promising cell sources for the regeneration or reconstruction of various somatic tissues, including those in the cranio-maxillofacial region. Thus, stem cell biology using these cells has become an important research field for understanding and implementing regenerative medicine.

The aim of this review was to summarize and compare the features of DPSCs and SHEDs, and their potential to regenerate damaged tissues, as well as future prospects of cell reprogramming application for cell-based regenerative therapies.

## 2. Self-Renewal and High Growth Capacity of DPSCs and SHEDs

**Figure 2:**
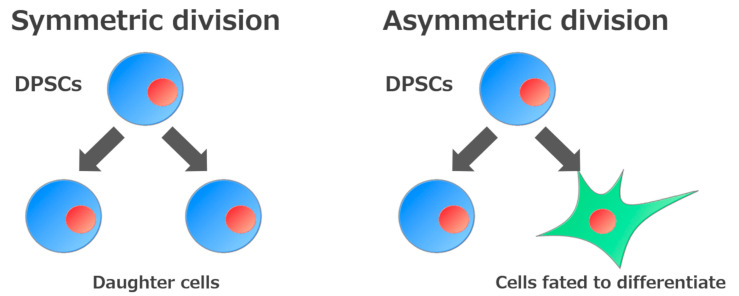
Self-renewal mechanisms of stem cells. Stem cells are defined by their ability to make new stem cells (self-renewal). Self-renewal of stem cells is divided into two processes: Generation of daughter cells or of cells fated to differentiate.

The ability to self-renew is one of the essential defining features of stem cells. This ability is divided into two types according to how they self-renew. One involves generating daughter cells with developmental potential similar to that of the original stem cells (symmetric division), while the other involves generating both undifferentiated cells and cells fated to differentiate (asymmetric division) [18] (Figure 2). Consequently, stem cells can expand during development, be maintained in somatic tissues, and participate in tissue regeneration.

DPSCs have been clarified to show robust proliferative activity. Gronthos et al. revealed that single-colony-derived strains of human DPSCs could proliferate beyond 20 population doublings [14]. Mokry et al. also measured population doublings of 11 different human DPSC lines [19]. Interestingly, all lines were capable of growing beyond Hayflick’s limit and the highest population doubling number was 81. These results strongly suggested the high self-renewal ability of human DPSCs. Bone-marrow-derived mesenchymal stem cells (BMMSCs) possess high self-renewal potential, so they are regarded as a promising cell source for biomedical research, drug discovery, and cell-based therapies. A previous study compared colony-forming efficiency and cell proliferation between human DPSCs isolated from extracted third molars of adults (19–29 years of age) and human BMMSCs derived from aspirated bone marrow cells of adults (20–35 years of age) [9]. Surprisingly, human DPSCs showed a higher frequency of colony-forming cells and a greater number of proliferating cells than human BMMSCs. Additionally, another study investigated the self-renewal properties of human DPSCs and BMMSCs derived from donors of the same age (18–25 years) [20]. The results showed that these DPSCs also exhibited higher proliferative and colony-forming potential, compared with BMMSCs. Moreover, Alge et al. compared the self-renewal capacity between rat DPSCs and BMMSCs using a donor-matched experimental design [21]. In that experiment, DPSCs had a shorter doubling time and formed more colonies and clones than BMMSCs. Given these results, the self-renewal ability of DPSCs is suggested to be greater than that of BMMSCs. A possible explanation for this is the activity of a ribonucleoprotein DNA polymerase complex, telomerase. Undergoing repeated cell divisions induces a significant shortening of telomeres, which triggers cellular senescence and proliferation arrest; however, telomerase compensates for their shortening in stem cells [22]. A previous study revealed a higher expression level of telomerase in human DPSCs than that in human BMMSCs [13]; therefore, telomeres in human DPSCs would be maintained at greater lengths than those in human BMMSCs via their high telomerase activity. These results confirm the robust proliferative activity of DPSCs, indicating that they can maintain their self-renewal activity even if they are further cultivated to obtain enough cells for clinical use.

SHEDs were reported to exhibit a higher proliferation rate than human DPSCs [10]. Nakamura et al. demonstrated that human SHEDs highly expressed several genes associated with pathways related to cell proliferation, such as *fibroblast growth factor 2 (FGF2)* and *transforming growth factor-β* (*TGF-β*), compared with human DPSCs. In addition, SHEDs exhibited higher expression of stemness-related markers such as Sox2 and Nanog compared with DPSCs [16], suggesting their more immature state than DPSCs. These results might also be explained by a critical shortening of telomeres due to their iterated cell divisions. 

Various signaling pathways like platelet-derived growth factor-activated signaling, hepatocyte growth factor-activated signaling, epidermal growth factor-activated signaling, and TGF-β-activated signaling are involved in regulating the self-renewal properties of stem cells [23]. Similar to the case in other types of stem cell, the involvement of several types of signaling in the proliferation of DPSCs has been reported. For example, the Notch–Delta1 signaling pathway was found to be associated with the proliferative and colony-forming potential of human DPSCs [24]. In addition, Wingless-type MMTV integration site family, member 10A (Wnt10A), and tumor necrosis factor alpha (TNF-α) enhanced the proliferation of human DPSCs via activation of the WNT/β-catenin signaling pathway and AKT/GSK-3β/Cyclin D1 signaling pathway, respectively [25,26]. Intraflagellar transport 80 (IFT80) was also shown to play crucial roles in the proliferation of mouse DPSCs via regulating the FGF–PI3K–AKT signaling pathway [27]. Moreover, transient receptor potential melastatin 4 channel was revealed to be involved in the proliferation and survival of rat DPSCs by controlling intracellular Ca^2+^ signals [28]. Furthermore, Gao et al. demonstrated that the growth capacity of PDLSCs was associated with JNK and p38 MAPK pathways, whereas the proliferation of DPSCs appeared to be dependent on ERK1/2 MAPK pathway activation [29]. However, the precise signaling cascade regulating the proliferation and self-renewal of DPSCs has not been clarified. To evaluate the precise signaling cascades, analysis of the effects of the combined use of growth factors and specific signal inhibitors on the proliferation of DPSCs will be helpful for researchers to understand their signaling interactions. Further studies on the interaction between these signaling cascades involved in the proliferation and self-renewal ability of DPSCs should be helpful to expand and prepare sufficient DPSCs for therapeutic application.

It is clear that hypoxia plays fundamental roles in the self-renewal properties of human embryonic, hematopoietic, mesenchymal, and neural stem cells. As dental pulp tissue is surrounded by dentin and enamel, for its oxygen, it depends on the supply through capillary blood vessels. Oxygen tension in dental pulp tissue is lower than that in cell culture conditions because in vitro cell cultures are usually maintained in a humidified atmosphere with 5% CO_2_. It has been reported that oxygen tension in rat dental pulp tissue was 23.2 mmHg (approximately 3% O_2_) [30,31]. Concerning the clinical application of DPSCs for the regeneration of dentin/pulp complex by cell transplantation, it may be important to analyze the effects of hypoxic culture conditions that reflect the in vivo environment. Some researchers investigated the promotive effect of hypoxia on the proliferation and colony formation of human DPSCs and SHEDs [31,32]. Kwon et al. demonstrated that hypoxic conditions increased the proliferation rate of DPSCs compared with the level of those cultured under normoxic conditions [33]. In contrast, some studies demonstrated that hypoxia did not change their proliferation and survival [34,35]. As such, the effect of hypoxia on the self-renewal ability of DPSCs and SHEDs is still unclear and further research is needed to clarify their regulatory mechanisms under hypoxic conditions.

## 3. Multipotency of DPSCs and SHEDs

**Figure 3:**
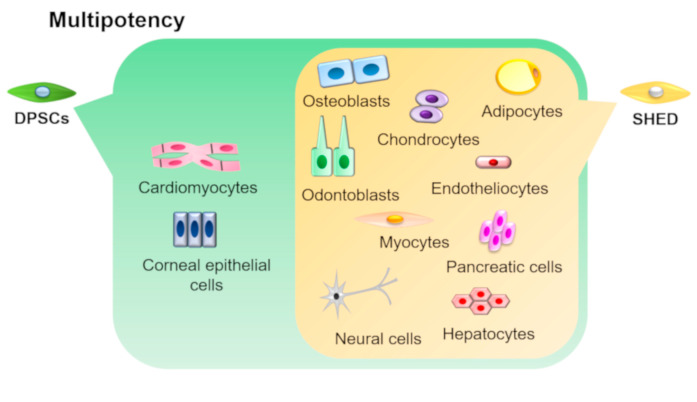
Multipotency of DPSCs and SHEDs. DPSCs and SHEDs can differentiate into multiple lineages such as osteoblasts, odontoblasts, adipocytes, chondrocytes, neural cells, endotheliocytes, myocytes, hepatocytes, and pancreatic cells under appropriate culture conditions. In addition, DPSCs can also differentiate into corneal epithelial cells and cardiomyocytes.

DPSCs and SHEDs have the ability to differentiate into various cell types under appropriate culture conditions (Figure 3).

Previous studies revealed that DPSCs and SHEDs have the potential to undergo osteo/odontogenic differentiation [36,37]. In addition, several in vivo studies demonstrated that DPSCs and SHEDs could differentiate into odontoblast-like cells and formed dentin/pulp complexes when they were transplanted subcutaneously into the dorsal surface of immunocompromised mice [10,38,39]. This suggested that they may be able to differentiate into odontoblasts in vitro and in vivo.

As for osteo/odontogenic potential, DPSCs and SHEDs have been reported to differentiate into adipocytes, chondrocytes, neural cells, endotheliocytes, myocytes, hepatocytes, and pancreatic cells [10,14,40,41,42,43,44,45,46,47,48]. Moreover, DPSCs have also been proven to differentiate into cardiomyocyte-like cells when they were co-cultured with neonatal rat cardiomyocytes in vitro [49]. Additionally, Gomes et al. fabricated a cell sheet of DPSCs and transplanted it onto injured rabbit cornea. Their results showed that the transplanted DPSC sheets integrated into the ocular tissue and reconstructed the corneal epithelium at 3 months after transplantation [50], suggesting that DPSCs have the ability to differentiate into corneal epithelial cells. Recently, many reports on the neurogenic differentiation of DPSCs have been published. For example, Karbanová et al. reported that DPSCs could differentiate into neural cells by the formation of neurospheres [51]. In addition, Xiao et al. showed that DPSCs could differentiate into glial cells as well as neural cells [52]. Furthermore, DPSCs cultured in neurogenic medium produced a sodium current just like functionally active neurons [53]. Although DPSCs exhibited better neurocytic differentiation than SHEDs, SHEDs had better osteogenic and adipogenic differentiation abilities than DPSCs [16]. In so far as regeneration of neural tissues, DPSCs might be a better cell source than SHEDs, whereas SHEDs exhibit superior therapeutic effects on regeneration of bone and adipose tissues compared with DPSCs. Numerous comparative studies using animal models to evaluate their regenerative potential will be helpful for clinicians when making choices for cell source in clinical situations. To summarize the multipotency of DPSCs and SHEDs, further clarification of their differentiation ability could make them indispensable cell sources for the regeneration of various somatic tissue.

## 4. Cell Markers Expression in DPSCs and SHEDs

In general, DPSCs and SHEDs express a variety of markers similar to BMMSCs (Table 1). DPSCs express MSC-related markers, such as CD13, CD44, CD73, CD90, CD146, CD166, and STRO-1 [9,16,54,55,56,57,58,59,60,61,62], while they do not express monocytic and hematopoietic lineage markers, such as CD14, CD19, CD34, CD45, and HLA-DR surface molecules [9,16,55,56,63]. SHEDs have been reported to show an expression pattern similar to DPSCs, such as CD13, CD44, CD73, CD90, CD146, CD166, and STRO-1 [10,16,17,56,57,61,62], but not CD34, CD45, or HLA-DR [16,17,56,57]. The absence of these monocytic and hematopoietic lineage markers is a defining feature of mesenchymal cells. Like ES cells, DPSCs and SHEDs have also been shown to express markers of pluripotency, such as Oct-4, Nanog, Sox-2, and insulin-like growth factor 1 receptor (IGF1R) [16,17,56,64]. Previously, it has been reported that SHEDs showed higher expression of pluripotent markers such as Oct-4, Nanog, and Sox-2 than DPSCs [16]. These results may reflect the notion that SHEDs exhibit a higher proliferation rate than DPSCs, indicating their more immature state [10]. In addition, under basal conditions, DPSCs and SHEDs express osteogenic markers, such as bone morphogenetic protein 2 (BMP2), osteocalcin (OCN), osteopontin (OPN), osteonectin, and type 1 collagen (Col-1) [9,17,58,65]; adipogenic markers, such as peroxisome proliferator-activated receptor gamma (PPARγ) and lipoprotein lipase (LPL) [10,14,17,58,66]; chondrogenic markers, such as type 2 collagen (Col-2) and Sox9 [17,58]; myogenic markers, such as alpha smooth muscle actin (α-SMA), myogenin, and myosin [9,17,58,67]; neurogenic markers, such as nestin, glial fibrillary acidic protein (GFAP), β-III tubulin, and microtubule-associated protein 2 (MAP-2) [14,16,17,56,58,63,68,69]. These results support the assertion that they have MSC-like properties including pluripotency and multipotency, indicating their protective effects for regenerative medicine.

Because of the heterogeneity of DPSCs, no specific markers for identifying these cells have been discovered. As mouse incisors continuously grow throughout the lifetime, they provide an excellent tool for analyzing specific markers and their contribution to the development and regeneration of dentin/pulp complex. Some researchers investigated novel specific markers to define DPSCs using a mouse incisor model. For example, Feng et al. demonstrated that cells with positivity for NG2, a proteoglycan used as a marker of pericytes [70,71], in pulp tissue could differentiate into polarized odontoblasts in response to postnatal growth and injury of mouse incisor [72]. Zhao et al. also reported that NG2-immunoreactive cells were increased in odontoblasts at 3 weeks after incisor injury, indicating their contribution to reparative dentin formation [73]. Although pericytes with positivity for NG2 show myogenic potential [74,75], these results suggested that NG2 could also be a marker for DPSCs. In addition, An et al. reported that CD90^+^ MSCs could form dentin/pulp complexes during postnatal development and wound healing of mouse incisor [76]. Moreover, Matsui et al. isolated CD146^+^ DPSCs using magnetic-activated cell sorting and transplanted them into immunodeficient mice [77]. Their results showed that CD146^+^ DPSCs promoted mineralization and generated dentin/pulp complex. Magnetic separation using specific antibodies against cell surface markers such as CD90 and CD146 can be helpful to analyze their commitment to cellular differentiation and tissue regeneration. Further studies using rat incisor models or magnetic cell sorting should be helpful to analyze the properties of cell markers that DPSCs and SHEDs express and to clarify novel specific markers of DPSCs and SHEDs. In addition, they might help researchers and clinicians to isolate purified stem cells in a clinical setting for regenerative medicine.

Previously, it has been reported that SHEDs showed higher expression of pluripotent markers such as Oct-4, Nanog, and Sox-2 than DPSCs [16]. However, Kanafi et al. revealed that there was no difference in the expression levels of CD73 and CD90 between DPSCs and SHEDs, but SHEDs showed higher expression of CD105 than DPSCs [61]. On the other hand, Gonmanee demonstrated that DPSCs showed higher expression of CD73 and CD105 than SHEDs, whereas the expression of CD90 was higher in SHEDs than in DPSCs [62]. Moreover, Suchánek et al. reported that SHEDs presented higher expression of CD105 and CD166 than DPSCs [78]. Further comparative studies about the marker expression in DPSCs and SHEDs will be helpful for precise characterization and identification of these cells. 

## 5. Immunomodulatory Effects of DPSCs and SHEDs

Although autologous DPSCs and SHEDs are considered appropriate cell sources for cell-based medicine, it is often difficult to generate enough cells from one donor because their biological activities such as proliferation and differentiation vary between donors. In contrast, the use of allogeneic cells may lead to immune rejection by the host’s immune system due to major histocompatibility complex mismatch. However, to date, MSCs have been shown to exhibit immunomodulatory abilities through the expression of enzymes, the production of soluble factors, and cell-to-cell contacts [79,80,81]. Previous studies reported immunomodulatory properties of DPSCs in co-culture models using Transwells with immune cells, such as T cells and peripheral blood mononuclear cells (PBMCs) [82,83,84]. In these studies, the immune cells were stimulated with different enzymes or soluble factors, such as concanavalin A (Con A), phytohemagglutinin (PHA), and anti-CD3/CD28 antibodies, to activate their proliferation and differentiation. DPSCs suppressed the proliferation of Con A-treated PBMCs in co-cultures using Transwells [83]. Co-culturing of DPSCs and PHA-activated T cells inhibited T cell proliferation and induced T cell apoptosis and the formation of regulatory T cells (Tregs) which play a central role in immune homeostasis [82,85,86]. In addition, DPSCs inhibited the proliferation and cytokine production of CD4^+^ T cells, but promoted differentiation into Tregs [84]. Kwack et al. reported that co-culturing of DPSCs and anti-CD3/CD28 antibody-treated PBMCs inhibited CD8^+^ T cell proliferation [87]. Their results indicate that DPSCs exhibit immunomodulatory effects by inhibiting the proliferation of T cells probably via Fas ligand (FasL) [88], but promoting Treg differentiation.

In addition to inhibitory effects on the proliferation of T cells, DPSCs upregulated the secretion of anti-inflammatory factors, such as IL-6, TGF-β, hepatocyte growth factor (HGF), and IL-10, in T cells, whereas they downregulated the production of pro-inflammatory factors, such as IL-2, IL-12, and TNF-α [85]. DPSCs have also been proven to decrease the expression of interferon gamma (IFN-γ), IL-4, and IL-17A but increased the expression of IL-10 and TGF-β in CD4^+^ T cells [84]. In contrast, DPSCs also achieve immunomodulatory effects via the expression of soluble factors that regulate the functions of immune cells. It has been reported that some immunomodulatory and anti-inflammatory factors, such as IL-6, IL-8 TGF-β, HGF, and indoleamine 2,3-dioxygenase (IDO) were expressed in DPSCs [89,90]. IL-6 and TGF-β have been reported to increase M2 macrophage polarization, which contributes to the clearing of apoptotic cells and mitigation of the inflammatory response [91,92]. DPSCs promoted M2 macrophage polarization when they were transplanted into the unilateral hindlimb skeletal muscle and suppressed sciatic nerve inflammation [93], indicating that DPSCs regulate M2 macrophage activation probably via expression of immunomodulatory and anti-inflammatory factors. DPSCs have also been reported to release TGF-β in response to nerve injury and to facilitate nerve repair and regeneration by inhibiting acute immune response [87,94]. These findings suggest that DPSCs exhibit their immunomodulatory properties not only by regulating the proliferation and cytokine production of immune cells, but also via the expression of immunomodulatory and anti-inflammatory factors in DPSCs.

SHEDs also exhibit immunomodulatory functions, like DPSCs. Dai et al. seeded PBMCs onto cultured SHEDs in the presence of PHA and demonstrated that SHEDs inhibited the proliferation of T cells and decreased the expression of pro-inflammatory factors, such as IL-4 and IL-17A, but increased the expression of anti-inflammatory factors, such as IL-10 and TGF-β, in PHA-activated PBMCs [95]. SHEDs also affected the immune response of monocyte-derived dendritic cells (DCs). When DCs were seeded onto cultured SHEDs, DCs decreased the expression of pro-inflammatory factors, such as IL-2, TNF-α, IFN-γ, while increasing the expression of IL-10 [96]. In addition, Yamaza et al. reported that SHEDs inhibited CD4^+^ T cells to differentiate into T helper 17 cells (Th17) when CD4^+^ T cells were loaded on the SHEDs culture [97]. Several in vivo studies also proved the immunomodulatory effects of SHEDs. For example, SHEDs transplantation had therapeutic effects on systemic lupus erythematosus-like diseases through the upregulation of Treg formation, and on ovariectomy-induced osteoporosis via activation of T cell apoptosis and the inhibition of Th1 and Th17 formation in mice [97,98]. Gao et al. also reported that the local delivery of SHEDs suppressed periodontal tissue inflammation and induced M2 macrophage polarization in a rat periodontitis model [99]. In addition, SHEDs transplantation improved the hyposalivation caused by Sjögren’s syndrome through downregulating Th1/Th2/Th17-related cytokine expression in rats [100]. Furthermore, SHEDs exhibited superior immunomodulatory effects compared with BMMSCs in terms of inhibiting Th17 activity and Th2 immune response, and promoting Treg formation [95,97]. Their results suggested that SHEDs show immunomodulatory effects by inhibiting the proliferation and differentiation of immune cells and regulating the expression of pro- and anti-inflammatory factors in those cells in a cell-cell contact manner.

Recently, extracellular vesicles (EVs), which are classified into exosomes and microvesicles (i.e., bilayer membrane structures transferring a range of bioactive components such as RNA, protein, and lipids), have reported to show immunomodulatory properties [101,102]. Thus, investigation of the effects of EVs from DPSCs and SHEDs on immune cells such as T cells and macrophages, and analysis of the components of such EVs might lead to clarification of the precise mechanisms behind their immunomodulatory effects. This could also confer potential for the transplantation of autologous and allogeneic cells in regenerative medicine, although this requires further study.

To summarize immunomodulatory properties of DPSCs and SHEDs, these cells exhibit immunomodulatory effects by regulating proliferation and cytokine production of immune cells. DPSCs also express immunomodulatory and anti-inflammatory factors by themselves to regulate the activation of immune cells. Although whether SHEDs express such factors remains unclear, they can exhibit immunomodulatory effects in a cell-cell contact manner.

## 6. Regenerative Capacity of DPSCs and SHEDs

The most notable feature of DPSCs and SHEDs is their odontoblastic differentiation ability. In the case of irreversible pulp inflammation, dental pulp tissue is extracted and root canal filling is performed using biocompatible materials. However, endodontically treated teeth are weakened due to dental caries and subsequent prosthodontic treatment, and are at increased risk of root fracture, eventually requiring tooth extraction [103,104]. Because dental pulp tissue plays a crucial role in tooth homeostasis, viable pulp is essential to extend the lifetime of the tooth [105]. Thus, a goal of endodontic treatment is the complete regeneration of dentin/pulp complex including tubular dentin formation, pulp innervation, pulp immunity, and vascularization. 

### 6.1. Regeneration of Dentin/Pulp Complex and Other Dental Tissues

Previous reports suggested that the autologous transplantation of DPSCs or SHEDs may be a safe and promising approach for dentin/pulp regeneration [106,107]. The general procedure of regenerative endodontics is transplantation of these cells with biocompatible scaffolds or porous biomaterials. DPSCs mixed and subcutaneously transplanted with hydroxyapatite/tricalcium phosphate (HA/TCP) or nanofibrous poly-L-lactic acid (PLLA) differentiated into odontoblasts and formed dentin including vascularized pulp-like tissue in immunodeficient mice [9,14,108,109,110,111]. In addition, DPSCs mixed with fibrin or TCP also showed the formation of dentin-like tissue when they were transplanted subcutaneously or under the renal capsule of mice [112,113]. Because of their lineage specific propensity, DPSCs could be a promising cell source for dentin/pulp complex and provide favorable therapeutic effects.

SHEDs have also been reported to possess the ability to regenerate dentin/pulp complex, like DPSCs. For example, Miura et al. revealed that SHEDs subcutaneously transplanted with HA/TCP formed dentin in immunocompromised mice [10]. Other studies revealed that SHEDs subcutaneously transplanted with hydrogel, collagen type 1, or PLLA into immunocompromised mice formed pulp-like tissues including odontoblasts and blood vessels [114,115]. However, SHEDs could not achieve the complete reconstruction of dentin/pulp complex in vivo [116]. Miura et al. also reported that only 3 of 12 single-colony-derived SHED clones formed dentin-like tissue [10]. Their results suggest that SHEDs contain subpopulations of cells that can differentiate into odontoblasts. Although numerous studies have revealed the availability of SHEDs for dentin/pulp regeneration as we described above, further clarification of the features of subpopulations of SHEDs is needed to translate them into a clinical setting. At present, DPSCs may be an appropriate cell source for regeneration of dentin/pulp complex compared with SHEDs.

Many researchers have relied on the use of biomaterials or scaffolds in which cells can proliferate and differentiate for various purposes, but the most appropriate scaffold as a carrier for cell transplants has not been identified. Despite conventional scaffold-mediated methods being available, the use of scaffold-free methods in regenerative endodontics has been reported. For example, Dissanayaka et al. prepared spheroids of DPSCs prevascularized by human umbilical vein endothelial cells and implanted them with a tooth slice into the subcutaneous space of the dorsum of immunodeficient mice [117]. The results showed the regeneration of well-vascularized pulp-like tissues. In addition, it has been reported that scaffold-free three-dimensional constructs of DPSCs regenerated pulp-like tissue with abundant blood vessels after their implantation into immunodeficient mice [118]. These scaffold-free cell constructs could be a novel approach for cell-based therapy because the use of scaffolds is associated with the risks of infection and inflammation. 

Several studies have been conducted to clarify the effects of DPSCs and SHEDs in the regeneration of periodontal tissue. For example, Khorsand et al. transplanted DPSCs with bone granules into periodontal defects in a canine periodontitis model and revealed that DPSCs regenerated cementum and periodontal ligament (PDL) tissue [119]. In addition, Janebodin et al. reported that DPSCs enhanced the differentiation of human salivary gland (HSG) cell into functional salivary gland tissue when they were mixed and subcutaneously transplanted into immunocompromised mice [120]. Furthermore, Otaki et al. investigated the effects of DPSCs on bone formation when they were subcutaneously transplanted with HA/TCP powder into immunocompromised mice. At 15 weeks after transplantation, the DPSCs formed bone tissue including lamellar bone on the surface of HA/TCP [121]. Other studies also reported the bone regenerative effects of DPSCs [122,123]. Although SHEDs have also been reported to induce bone formation in rodents [124,125] and swine [126], Miura et al. revealed that they could not differentiate directly into osteoblasts, but recruited host osteogenic cells and induced them to become osteoblasts [10]. It is still obscure whether SHEDs promote the osteoblastic differentiation of host osteogenic precursors by producing growth factors or via cell-to-cell contacts. Further clarification of the factors released from SHEDs and studies using co-culture models of SHEDs and osteogenic precursors might help to understand the precise mechanism involved.

### 6.2. Regeneration of Other Somatic Tissues

In addition to the regeneration of dental tissues, DPSCs have been reported to possess the ability to regenerate or repair various somatic disorders such as cornea trauma [127], glaucoma [128], muscular dystrophy [129], acute myocardial infarction [130], spinal cord injury [131], liver fibrosis [132], cerebral ischemia [133], diabetic neuropathy [134], and osteoporosis [135]. SHEDs have also been reported to exhibit regenerative potential against spinal cord injury [66], hypoxic–ischemic brain injury [136], systemic lupus erythematosus [98], ulcerative colitis [137], and liver fibrosis [138,139]. The isolation of DPSCs and SHEDs is less invasive than that of MSCs obtained from other somatic tissues, and the therapeutic effects of these cells have been well investigated using various extraoral disease models. Thus, DPSCs and SHEDs have been considered particularly attractive as cell sources for the regeneration of not only dental tissue but also other somatic tissues.

### 6.3. Cell-free Methods for Regenerative Medicine

It has been shown that conditioned medium obtained from MSCs has therapeutic effects in several somatic disorders [140,141]. Conditioned medium from DPSCs and SHEDs have also been clarified to have regenerative potential [142,143,144,145]. Although the main substance contained in conditioned medium and exerting therapeutic effects has not been clarified, exosomes might play an important role in regenerative processes. Exosomes derived from DPSCs increased the odontogenic differentiation of DPSCs [146] and promoted the regeneration of dental pulp-like tissue [147]. In addition, Li et al. demonstrated that exosomes derived from SHEDs suppressed neuroinflammation when they were transplanted into a rat brain injury model [148]. The use of conditioned medium or exosomes has advantages over cell transplantation methods using biomaterials or scaffolds because of the avoidance of immune rejection and embolus formation. Further studies on the therapeutic effects of conditioned medium and EVs such as exosomes or microvesicles using in vivo models might lead to their use as alternative agents for cell-free regenerative medicine.

#### 6.3.1. Exogenous Growth Factors

Cell transplantation methods are at risk of immune rejection, tumorigenesis, and pathogen transmission. As opposed to cell transplantation, the stem cell homing strategy, which involves the recruitment of the patients’ endogenous stem or progenitor cells, has attracted more attention. Thus, identification of growth factors that direct cell homing ability will be proof of the efficiency of cell homing methods in regenerative medicine. Cell homing includes three different cellular processes: Cell migration, proliferation, and differentiation [149]. As for cell migration, Granulocyte-colony stimulating factor (G-CSF) [150,151], stromal cell-derived factor-1 (SDF-1) [151,152], TGF-β1 [153], and basic fibroblast growth factor (bFGF) [154] can induce the migration of DPSCs. On the other hand, Wnt3A [155], bFGF [156], G-CSF [150], and SDF-1 [151] have been reported to exhibit the proliferative effect on DPSCs. Furthermore, considering the differentiation, BMP2 [157], BMP7 [154], and G-CSF [150] induced odontoblastic differentiation of DPSCs. Their results suggest that G-CSF may be a potent factor for cell homing-based therapy. Further studies are needed to validate either the use of single growth factor or a subset of growth factors would be effective for cell homing.

The underlining mechanisms of stem cells state or differentiate is mainly due to epigenetic regulation because DNA methylation and histone modification regulate the MSC differentiation [158]. Among epigenetic regulators, histone deacetylases (HDACs) which regulates the structure of chromatin and transcription have been reported to play an important role in cell differentiation and the developmental process [159]. Paino et al. reported that valproic acid (VPA), a HDAC inhibitor, improved mineralized matrix formation and enhanced the expression of bone-related genes in DPSCs [160]. In addition, DPSCs treated with VPA produced a well-organized lamellar bone tissue formation when they were subcutaneously transplanted into immunocompromised mice [158]. Moreover, HDAC inhibitors, such as trichostatin A and suberoylanilide hydroxamic acid, have also been proven to improve self-renewal [161] and migration [162] abilities of DPSCs. Therefore, application of such epigenetic regulators may be an alternative approach for tissue regeneration which can intervene in stem cell activities. Further animal trials should be performed to validate the cell homing method using growth factors or epigenetic regulators as an alternative approach for tissue regeneration before human trials.

#### 6.3.2. Semaphorin 3A

Semaphorin 3A (Sema3A), which is a member of the semaphorin family, was identified as an axonal guidance factor regulating nervous system development [163], and has been reported to play important roles in the development of various tissues such as blood vessels, peripheral nerves, and skeletal tissues [164,165,166]. In addition, Sema3A acts as an osteoprotective factor by inhibiting osteoclastic bone resorption and promoting osteoblastic bone formation [167]. Our previous study demonstrated that a multipotent human PDL cell line [168] highly expressed Sema3A compared with a PDL cell line with low differentiation potential (low-potent) [169]. In addition, we generated a Sema3A-overexpressing low-potent PDL cell line and clarified that these cells exhibited enhanced capacity to differentiate into osteoblasts and adipocytes. Moreover, Sema3A-overexpression enhanced self-renewal ability and increased the expression levels of markers of pluripotency, such as Oct-4, Nanog, and E-cadherin, and; MSC-related markers, such as CD73, CD90, CD105, CD146, and CD166 [169]. Our results indicate that Sema3A regulates the stem cell features of PDLSCs and induction of this gene provide multipotency for a low-potent PDL cell line. Yamada et al. reported that Sema3A knockdown decreased self-renewal ability and the expression of Sox2, Oct-4, and CD44 in lung cancer stem cells [170]. Their results support our hypothesis that Sema3A would play an important role in acquisition and maintaining of stemness. 

**Figure 4:**
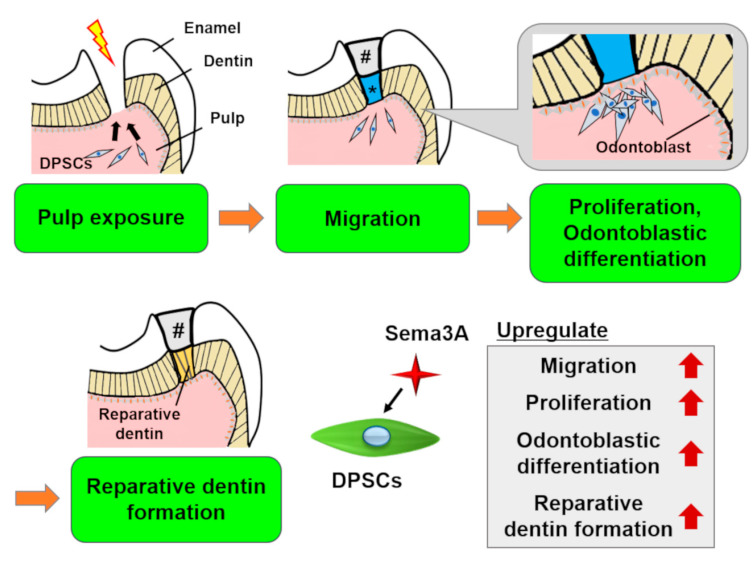
Schema of the effects of Semaphorin 3A (Sema3A) on DPSCs. In case of pulp exposure, DPSCs migrate toward the exposure site, then they proliferate and differentiate into odontoblasts to form reparative dentin. Sema3A can act as a bioactive agent to facilitate these regenerative processes. #, sealing material; *, Sema3A. The original data was published in [40].

Recently, we demonstrated that Sema3A increased migration, proliferation, and odontoblastic differentiation of DPSCs [40] (Figure 4). Moreover, Sema3A facilitated reparative dentin formation after direct pulp capping in a rat pulp exposure model. These results suggested that Sema3A can recruit latent DPSCs to the wounded site and increase its biological activities to regenerate dentin/pulp complex. At the present time, we are planning to clarify the precise mechanisms of epigenetic regulation by Sema3A and to confirm the possibility for its use as a therapeutic agent that could increase the activity of hosts’ latent progenitor cells, especially DPSCs. 

#### 6.3.3. Side Population Cells from Dental Pulp and Mobilized Dental Pulp Stem Cells

Previously, Iohara et al. successfully isolated CD31^-^/CD146^-^ side population (SP) DPSCs from porcine dental pulp tissue and revealed their high self-renewal ability, multilineage differentiation potential [171]. Thereafter, CD31^-^/CD146^-^ SP cells have been proven to promote migration and differentiation of endogenous neural progenitor cells, induce vasculogenesis, and ameliorate ischemia in hind limb and brain [151,172]. In addition, these cells also showed complete regeneration of dental pulp tissue with capillary blood vessels and neuronal cells when they were transplanted into a canine pulp amputation model [173]. Furthermore, Ishizaka et al. compared the regenerative potential of dental pulp, bone marrow, and adipose tissue-derived CD31^-^ SP cells, and demonstrated that CD31^-^ SP cells from pulp tissue promoted angiogenesis, neurogenesis, and pulp regeneration compared with those from bone marrow and adipose tissue [174]. These results suggest that SP cells from dental pulp such as CD31^-^/CD146^-^ and CD31^-^ SP cells can be better sources for treatment of ischemia diseases and pulp regeneration than those from other tissues.

Murakami et al. reported that DPSCs isolated by treatment of G-CSF exhibited enhanced anti-apoptotic activity, migration, immunomodulatory properties, and accelerated regeneration of pulp-like tissue [175]. G-CSF-treated DPSCs were defined as mobilized DPSCs (MDPSCs) and have been shown to exhibit their potential to regenerate well-vascularized dental pulp with rich nerve fibers [176,177]. Moreover, MDPSCs have also been demonstrated to regenerate dental pulp tissue in a pre-clinical human study [106]. These preferable results may be due to the properties of G-CSF that can induce proliferation, migration, and differentiation of DPSCs. In our previous study, Sema3A has also been shown to induce proliferation, migration, and odontoblastic differentiation of DPSCs [40]. Our results suggested that Sema3A-treated or Sema3A-overexpressing DPSCs may have the potential to regenerate dental pulp tissue as well as MDPSCs.

Overall, SP cells from dental pulp and MDPSCs showed therapeutic effects on pulp regeneration. Further randomized clinical trials and clarification of their therapeutic effects on other somatic tissues can provide their utility for regenerative medicine.

## 7. Cell Banking of DPSCs and SHEDs

Although DPSCs and SHEDs have regenerative activities in therapeutic applications, DPSCs from older patients or patients with systemic diseases such as diabetes, rheumatoid arthritis, or systemic lupus erythematosus show decreased bioactivity [178]. The isolation of SHEDs is impractical because the opportunity of the exfoliation is unpredictable. However, it has been reported that DPSCs and SHEDs can be cryopreserved to maintain their stemness and multipotency for many years [42,179,180]. Thus, cell banking by cryopreservation has attracted attention for the later use of such cells in clinical situations. Recently, cell banks for DPSCs and SHEDs have been established by some companies, such as BioEden^TM^ in the UK, Store-A-Tooth^TM^ in the USA, Cell Technology^TM^ in Japan. To be a promising cell source for regenerative medicine, further studies about the development of optimal banking procedure that does not affect the bioactivities of stem cells will be needed.

## 8. Conclusions

As reviewed in this article, DPSCs and SHEDs show MSC-like phenotypes such as self-renewal capacity, multipotency, MSC-related marker expression, and immunomodulatory effects. They also possess the capacity to regenerate various somatic tissues including dental tissues. However, there are several issues to address before the development of regenerative therapies using DPSCs and SHEDs. First, there is the need to understand the precise mechanisms of self-renewal ability of DPSCs and SHEDs, and to clarify the number of passages of these cells that are appropriate to induce tissue regeneration. In addition, specific molecular mechanisms and signaling cascades of soluble growth factors and exosomes derived from DPSCs and SHEDs are still under investigation. Controlling the activation of these factors may facilitate the cell differentiation and can contribute to achieving favorable tissue regeneration. Furthermore, as for the way to administrate DPSCs and SHEDs, which type of scaffold or the use of cell-sheets or three-dimensional constructs are the most applicable remain unclear. Moreover, understanding the interactions between these cells and the immune system are indispensable. Although DPSCs and SHEDs exhibit immunomodulatory effects, there is a need to determine how long these cells can suppress immune rejection by the host’s immune system. 

In 2007, Takahashi et al. first generated human induced pluripotent stem cells (iPSCs) by direct reprogramming with four genes, Oct-4, Sox2, Kruppel-like factor 4, and c-Myc [181]. iPSCs exhibit unrestricted proliferative potential and differentiation ability into three primary germ layer-derived cells, indicating that direct the reprogramming of somatic cells can provide the numerous immature cells required for cell-based medicine. Thereafter, the generation of iPSCs from DPSCs and SHEDs have been reported [182,183,184]. Xie et al. demonstrated that iPSCs derived from DPSCs formed dentin/pulp complex when they were transplanted subcutaneously into immunodeficient mice [183]. In addition, these cells differentiated into functionally odontoblasts regardless of their passage number, suggesting that they can cryopreserve for further use without the deletion of their differentiation ability.

Previously, we established PDLSCs from iPSCs using the extracellular matrix [185]. In that study, human skin fibroblast-derived iPSCs were inducted into neural crest-like cells, and these cells were further cultured on the extracellular matrix (ECM) produced by human PDL cells. They showed higher differentiation potential along with higher expression of MSC-related and PDL-related markers compared with neural crest-like cells. Induction of DPSCs from iPSCs using the ECM from dental pulp cells and their characterization have been under investigation. Our novel approach could be an alternative choice to acquire a large amount of DPSCs and SHEDs for clinical use; therefore, further studies are needed to clarify the features of these cells generated from iPSCs using ECM and to evaluate their regenerative potential in other somatic tissues. DPSCs and SHEDs established by the application of iPSCs or cell reprogramming factors that can convert matured dental pulp cells into immature cells may be used as promising and unlimited cell sources for the study of tissue engineering. Moreover, further clarification of their biological attributes and development of safe and more effective therapeutic approaches should facilitate their clinical use in regenerative medicine.

## Figures and Tables

**Table 1:** Expression of cell markers in DPSCs and SHEDs.

Marker Type	Expression Markers	DPSCs	SHEDs	Reference Numbers
**MSC-related**	CD13, CD44, CD73, CD90, CD146, CD166, STRO-1	**+**	**+**	[9,10,16,17,54,55,56,57,58,59,60,61,62]
**Osteogenic**	BMP2, OCN, OPN, Osteonectin, Col-1	**+**	**+**	[9,17,58,65]
**Adipogenic**	PPARγ, LPL	**+**	**+**	[10,14,17,58,66]
**Chondrogenic**	Col-2, Sox9	**+**	**+**	[17,58]
**Myogenic**	α-SMA, Myogen, Myosin	**+**	**+**	[9,17,58,67]
**Neurogenic**	Nestin, GFAP, β-III tubulin, MAP-2	**+**	**+**	[14,16,17,56,58,63,68,69]
**Pluripotent**	Oct-4, Nanog, Sox2	**+**	**++**	[16,17,56,64]
	IGF1R	**+**	**+**	
**Monocytic/hematopoietic**	CD14, CD19, CD34, CD45, HLA-DR	**-**	**-**	[9,16,17,55,56,57,63]

## Abbreviations

α-SMA	Alpha smooth muscle actin
bFGF	Basic fibroblastic growth factor
BMMSCs	Bone marrow-derived mesenchymal stem cells
BMP2	Bone morphogenetic protein 2
BMP7	Bone morphogenetic protein 7
Col-1	Type 1 collagen
Col-2	Type 2 collagen
Con A	Concanavalin A
DCs	Dendric cells
DFPCs	Dental follicle precursor cells
DPSCs	Dental pulp stem cells
ECM	Extracellular matrix
ERK	Extracellular signal-regulated kinase
EVs	Extracellular vesicles
FasL	Fas ligand
FGF	Fibroblast growth factor
GFAP	Glial fibrillary acidic protein
G-CSF	Granulocyte-colony stimulating factor
HGF	Hepatocyte growth factor
HDAC	Histone deacetylase
HSG	Human salivary gland
HA	Hydroxyapatite
IDO	Indoleamine 2,3-dioxygenase
iPSCs	Induced pluripotent stem cells
IGFR1	Insulin-like growth factor 1 receptor
IFN-γ	Interferon gamma
IL-2	Interleukin 2
IL-4	Interleukin 4
IL-6	Interleukin 6
IL-8	Interleukin 8
IL-10	Interleukin 10
IL-12	Interleukin 12
IL-17A	Interleukin 17A
IFT80	Intraflagellar transport 80
JNK	c-Jun N-terminal kinase
LPL	Lipoprotein lipase
MSCs	Mesenchymal stem cells
MAP-2	Microtubule-associated protein 2
MAPK	Mitogen-activated protein kinase
Oct-4	Octamer-binding transcription factor 4
OCN	Osteocalcin
OPN	Osteopontin
PDL	Periodontal ligament
PDLSCs	Periodontal ligament stem cells
PBMCs	Peripheral blood mononuclear cells
PPARγ	Peroxisome proliferator-activated receptor gamma
PI3K	Phosphatidylinositol-4, 5-bisphosphate 3-kinase
PHA	Phytohemagglutinin
PLLA	Poly-L-lactic acid
RNA	Ribonucleic acid
Sema3A	Semaphorin 3A
SDF-1	Stromal cell-derived factor 1
SCAPs	Stem cells from apical papilla
SHEDs	Stem cells from human exfoliated deciduous teeth
Sox2	SRY-box transcription factor 2
Sox9	SRY-box transcription factor 9
Th17	T helper 17 cells
TLRs	Toll-like receptors
TGF-β	Transforming growth factor-β
TCP	Tricalcium phosphate
TNF-α	Tumor necrosis factor alpha
Wnt10A	Wingless-type MMTV integration site family member 10A
